# Editorial: Glucosinolates: Regulation of Biosynthesis and Hydrolysis

**DOI:** 10.3389/fpls.2020.620965

**Published:** 2020-11-25

**Authors:** Bhanu Malhotra, Naveen C. Bisht

**Affiliations:** Brassica Nutrigenomics and Signaling lab, National Institute of Plant Genome Research, New Delhi, India

**Keywords:** glucosinolate, methylthioalkylmalate synthase, glucosinolate transporters, glucosinolate turnover, nitrile-specifier proteins, myrosinase, epithiospecifier proteins

The members of the *Brassicaceae* family possess a class of sulfur and nitrogen containing specialized metabolites glucosinolates (GSLs), that are important for plant defense and animal nutrition. Glucosinolates are both constitutive and inducible, and their accumulation is controlled by environmental, molecular, and genetic levels. Much of the information about the glucosinolate biosynthesis, transport, hydrolysis, turnover, crosstalk amongst biosynthesis and hormonal pathways, and mechanisms controlling glucosinolate accumulation and metabolism during abiotic and biotic stresses has been derived from the model plant *Arabidopsis thaliana* (Figure 1). However, there are still lacunae in fully understanding these regulatory processes in crop species. Thus, the purpose of this Research Topic is to decipher several aspects under the glucosinolate regulatory mechanisms, in view of which a total of eight research articles have been published.

**Figure 1:**
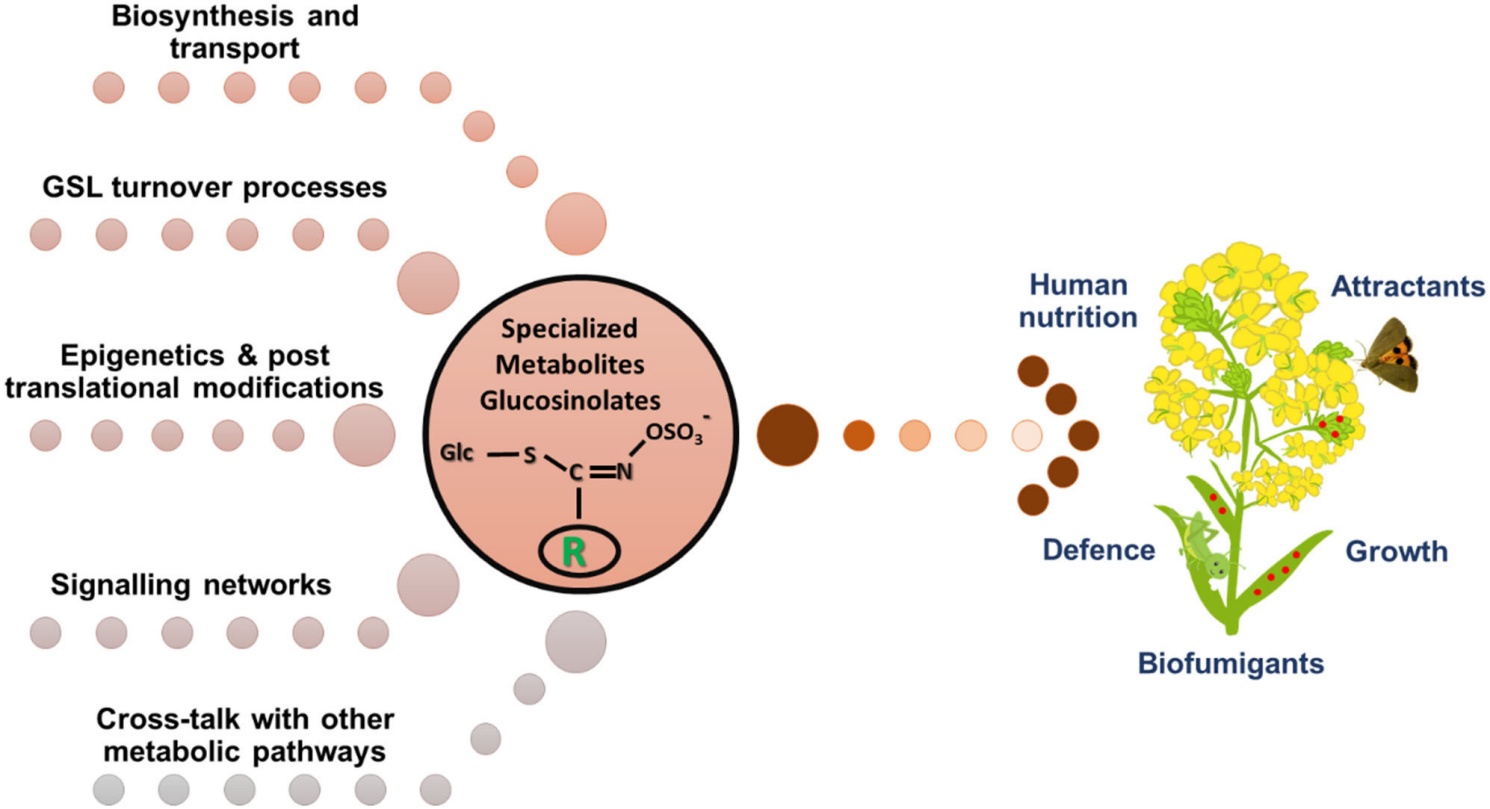
Regulation of glucosinolate metabolism in plants is a complex interplay among various biological processes.

Plants possess huge structural diversity of glucosinolates and studies in the last two decades have highlighted the key role of *GSL-ELONG* locus, encoding methylthioalkylmalate synthase (MAM or MAMS), controlling glucosinolates side chain diversity (3C, 4C, 5C) across the *Brassicaceae* species. Understanding the molecular basis of *MAM* evolution and generation of glucosinolate natural diversity in polyploid *Brassica* crops is a challenge due to their complex genome architecture (Kumar et al., 2019). Abrahams et al. investigated diversification of *MAM* locus utilizing a micro-synteny network and phylogenetic inference across a broader species phylogeny with comparisons to their primary metabolic ancestor, *isopropylmalate synthase*, uncovering critical steps in the origin of *MAM* and identifying patterns of domain specific diversity. With examination of few of the evolutionary consequences of whole-genome duplication events, local duplications, gene transpositions, and gene fusions, various new hypotheses are created to test the nature of *MAM* diversification and glucosinolate diversity in plants. The understanding of *MAM* evolution will enable fine engineering of desirable glucosinolate profiles in plants.

The accumulation of glucosinolates is controlled by the complex interplay of biosynthesis, transport, storage, and feedback regulatory pathways (Figure 1) the complexity of which is not fully understood. Touw et al. found distinct glucosinolate accumulation and composition during root herbivory in *Brassica rapa* infested with specialist *Delia radicum*. The increase in the total glucosinolate in the taproot was majorly due to indole and benzyl glucosinolates. It was initially hypothesized that the expression of glucosinolate transporter genes in distal tissues led to glucosinolate accumulation in taproot and preceded the localized expression of *de-novo* biosynthetic gene expression in the taproot. However, it was found that local biosynthesis but not transport from above ground parts drives the accumulation of indole glucosinolates in the taproot to protect from root-herbivory. Future studies should involve the use of isotope labeled glucosinolate precursors and understanding their transport upon root herbivory, confirming the precise role of transporter proteins in tissue-specific glucosinolate accumulation. Another study by Sontowski et al. also investigated the effect of two specialist herbivores *D. radicum and D. floralis* on the expression pattern of genes involved in glucosinolate biosynthesis, transport, and hydrolysis during infestation of *B. rapa* varieties accumulating low and high root glucosinolate levels. Only minute response differences were observed in the contrasting glucosinolate varieties of *B. rapa* with no trade-offs occurring between the two in the roots compared to those reported in shoots (Rasmann et al., 2015). *B. rapa* low glucosinolate variety could not compensate for the improved defense responses even after attaining similar glucosinolate levels as high glucosinolate variety. Thus, glucosinolate based defenses can differ in plants and between different herbivores, depending upon their host specialization levels and feeding modes.

With the changing environmental conditions, plants modulate their metabolism toward carrying out a balanced resource investment to both the development and defense pathways. The levels of glucosinolates in the intact tissue are a result of interplay amongst their biosynthesis and however uncharacterized turnover processes in response to the changing environment. Jeschke et al. investigated glucosinolate turnover in the absence of tissue damage in *A. thaliana* seedlings under different nutrient availability conditions, stating that both biosynthesis and turnover processes coordinate to achieve glucosinolate levels. While limitation of sulfur and nitrogen negatively affected the *de-novo* accumulation of all classes of glucosinolates, the exogenous application of carbon sources had quantitative effects on aliphatic glucosinolates. Raphanusamic acid (RA), a breakdown metabolite formed potentially from all glucosinolate structures in-planta (Bednarek et al., 2009), was found to correlate with enhanced accumulation of endogenous glucosinolates only but not its turnover in *A. thaliana*. Thus, RA could represent a metabolic checkpoint to control the flux of the glucosinolate biosynthesis and turnover processes in response to both external and internal signals. Meier et al., with the use of T-DNA mutants of enzymes responsible for glucosinolate breakdown, provided clue toward involvement of β-glucosidases belonging to BGLU18-BGLU33 clade, nitrile-specifier proteins (NSPs) during different stages of seed maturation and germination processes as potential candidates involved in glucosinolate turnover processes. The turnover processes are subjected to different substrate specificities of the glucosinolate hydrolytic enzymes. In addition, to evaluate the components involved in the glucosinolate turnover pathways, ascertaining the tissue specific effects of turnover is important like for example, increased turnover in one tissue balanced by enhanced biosynthesis in the other, balancing the total glucosinolate levels. Therefore, the study by Meier et al. has opened various dimensions and involvement of numerous candidates for glucosinolate turnover in the seeds, which require an in-depth investigation in coming years.

The biological activities of glucosinolates are governed post hydrolysis by myrosinases (β-thioglucoside glucohydrolase, TGG), where some hydrolysis products have the capabilities to induce stomatal closure (Hossain et al., 2013). But the physiological processes which control the TGG-mediated stomatal activity remain elusive. For further insights into these processes, Zhang et al. raised overexpression transgenics in *Arabidopsis* using *TGG1* homolog from broccoli and found enhanced resistant against the bacterial pathogen *Pseudomonas syringae*, by accelerating stomatal closure and inhibition of stomatal reopening upon challenge. This work provided a first indication that the TGG1-regulated stomatal defense response moves via multiple phytohormone signaling pathways. The *BoTGG1* overexpression lines also displayed delayed flowering phenotype by promoting the expression of the floral repressor, *FLOWERING LOCUS C (FLC)*- an indicative of the cross talk of the glucosinolate pathway to modulate flowering time regulation. This study opens a new avenue for manipulation of classical myrosinases for engineering pathogen resistance and control of flowering time, which are important breeding objectives for accumulating higher yields in the cultivated *Brassica* species.

Glucosinolates hydrolytic forms epithionitriles are triggered upon the presence of epithiospecifier proteins (ESPs) in the medium, and only glucosinolates which possess a terminal double bond in their side-chain structure can form epithionitriles. A single gene in the model plant *A. thaliana* governs epithionitrile formation, however our knowledge so far remains limited in the crops of the *Brassicaceae* family, as they underwent a lineage-specific whole genome triplication event after their split from *Arabidopsis*. Witzel et al. recently identified three *ESP* homologs using *in-silico* analysis in *B. oleracea*. The BoESPs possess differential transcript profiles across tissue types and exhibited distinct substrate specificities toward seven tested glucosinolate structures, to shape the glucosinolates hydrolysis product diversity in *B. oleracea* genotypes. The BoESP activities were examined using functional complementation studies in *A. thaliana* accession lacking functional ESP, suggesting isoform-specific roles of BoESPs in glucosinolate breakdown. The formation of nitriles and epithionitriles have several notified effects particularly higher susceptibility against herbivores, and reducing the attractiveness for oviposition of specialists by attracting their natural enemies. Epithionitriles in the *Brassica* vegetables have a lower impact on aroma and pungency as desired for human consumption, as opposed to ITCs which are the most comprehensively characterized GHP molecules so far. We still lack the full understanding of the biological significance of nitriles and epithionitriles toward their roles in biotic interactions. Ting et al. recently established the involvement of nitriles in disease resistance during pathogen challenge using 3-butenenitrile (3BN) treatment in *Arabidopsis*. Interestingly, plants treated with 3BN showed alleviated leaf lesion symptoms when challenged using *Pectobacterium carotovorum* and *Botrytis cinerea* consequently reducing pathogen growth on leaves. Therefore, 3BN molecule is proposed to act as a DAMP for the induction of broad immune responses in *Arabidopsis*, involving a crosstalk between signaling pathways for NO, ROS, JA, and SA production in plants. Future studies should focus to identify the precise target pathogen recognition receptors that might bind 3BN for its action. Therefore, such studies provide essential information to alter and reduce ESP activities in planta correlating with an optimal defense output.

In conclusion, we perceive the studies published within this Research Topic “*Glucosinolates: Regulation of Biosynthesis and Hydrolysis*” expand our current understanding of regulatory molecular mechanisms of glucosinolate biosynthesis, hydrolysis, turnover, and regulation in plants.

## Author Contributions

All authors listed have made a substantial, direct and intellectual contribution to the work, and approved it for publication.

## Conflict of Interest

The authors declare that the research was conducted in the absence of any commercial or financial relationships that could be construed as a potential conflict of interest.
